# A High-Density Integrated DArTseq SNP-Based Genetic Map of *Pisum fulvum* and Identification of QTLs Controlling Rust Resistance

**DOI:** 10.3389/fpls.2018.00167

**Published:** 2018-02-15

**Authors:** Eleonora Barilli, María J. Cobos, Estefanía Carrillo, Andrzej Kilian, Jason Carling, Diego Rubiales

**Affiliations:** ^1^Institute for Sustainable Agriculture, CSIC, Córdoba, Spain; ^2^Diversity Arrays Technology Pty Ltd, University of Canberra, Canberra, ACT, Australia

**Keywords:** DArT, *Pisum fulvum*, genetic linkage map, QTL, rust resistance, *Uromyces pisi*

## Abstract

*Pisum fulvum*, a wild relative of pea is an important source of allelic diversity to improve the genetic resistance of cultivated species against fungal diseases of economic importance like the pea rust caused by *Uromyces pisi*. To unravel the genetic control underlying resistance to this fungal disease, a recombinant inbred line (RIL) population was generated from a cross between two *P. fulvum* accessions, IFPI3260 and IFPI3251, and genotyped using Diversity Arrays Technology. A total of 9,569 high-quality DArT-Seq and 8,514 SNPs markers were generated. Finally, a total of 12,058 markers were assembled into seven linkage groups, equivalent to the number of haploid chromosomes of *P. fulvum* and *P. sativum*. The newly constructed integrated genetic linkage map of *P. fulvum* covered an accumulated distance of 1,877.45 cM, an average density of 1.19 markers cM^−1^ and an average distance between adjacent markers of 1.85 cM. The composite interval mapping revealed three QTLs distributed over two linkage groups that were associated with the percentage of rust disease severity (DS%). QTLs *UpDSII* and *UpDSIV* were located in the LGs II and IV respectively and were consistently identified both in adult plants over 3 years at the field (Córdoba, Spain) and in seedling plants under controlled conditions. Whenever they were detected, their contribution to the total phenotypic variance varied between 19.8 and 29.2. A third QTL (*UpDSIV.2*) was also located in the LGIVand was environmentally specific as was only detected for DS % in seedlings under controlled conditions. It accounted more than 14% of the phenotypic variation studied. Taking together the data obtained in the study, it could be concluded that the expression of resistance to fungal diseases in *P. fulvum* originates from the resistant parent IFPI3260.

## Introduction

The cultivated pea (*Pisum sativum* L. subsp. *sativum*) is one of oldest domesticated crops. Since its domestication about 10,000 years ago it has been improved for important agronomic traits, being today the cool season grain legume most cultivated in Europe and the second in the world (Rubiales et al., 2015). However, pea yield is still relatively unstable due to its limited adaptability to a wide range of environmental conditions and its susceptibility to diseases and pests. Among the biotic stresses, the rusts have been acquiring more and more economic importance since the mid-eighties. *Uromyces pisi* (Pers.) Wint. is the main causal agent of pea rust in temperate regions (Barilli et al., 2009a,b) although in tropical and subtropical regions as India or China, it has been reported to be caused by the fungus *Uromyces viciae-fabae* (Pers.) J. Schröt [syn. *U. fabae* (Pers.) de Bary] (Singh and Sokhi, 1980; Kushwaha et al., 2006). In years of epidemic the rust causes the drying of leaves and severely affects the development of pods, with consequent yield losses that can reach up to 50% (Barilli et al., 2009a).

Unfortunately, there is little resistance available in the cultivated *P. sativum*, being insufficient to achieve an effective control. Only some levels of incomplete resistance have been identified after an exhaustive screening of *U. pisi* resistance in a large germplasm collection identifying the highest levels of resistance in *P. fulvum* Sibth. & Sm. (Barilli et al., 2009a,b,c). Not without difficulty, crosses between *P. sativum* and *P. fulvum* have been possible and, in fact, resistance genes to *Bruchus pisorum* and *Erysiphe pisi* have already been transferred from *P. fulvum* to *P. sativum* by natural hybridization (Fondevilla et al., 2007; Byrne et al., 2008). Similarly, resistance to other biotic stresses such as *Didymella pinodes* or *Orobanche crenata* has been identified in *P. fulvum*, and crosses have also been carried out to transfer the resistance genes to the cultivated pea (Rubiales et al., 2005, 2009; Fondevilla et al., 2008, 2017).

The use of modern breeding tools will facilitate the efficient exploitation of all the potential offered by *P. fulvum* for *P. sativum* breeding. DNA-based genetic markers provide powerful tools for the identification and localization of genes that control traits of agronomic importance, whose subsequent introgression into commercial varieties facilitates the advancement of plant breeding programs. Despite the important source of resistance provided by *P. fulvum* so far there has been relatively little research on the development and application of molecular genetic tools in wild peas compared to the best studied *P. sativum* in which several types of markers have already been related to several disease resistance regions (Pavan et al., 2013; Carrillo et al., 2014; Coyne et al., 2015; Sudheesh et al., 2015; Tayeh et al., 2015; Boutet et al., 2016).

The association of molecular markers with *U. pisi* resistance has been previously studied in the F_2_ population derived from the intraspecific cross between the *P. fulvum* accessions IFPI3260 × IFPI3251 (Barilli et al., 2010). A previous genetic map of 1283.3 cM that included 146 markers (144 RAPDs and 2 STSs markers) distributed in 9 linkage groups (LGs) was developed. It was observed that rust resistance was governed by a major QTL that explained 63% of the total phenotypic variation located in the LG 3 and flanked by the RAPD markers OPY11_1316_ and OPV17_1078_. To our knowledge, there is no other report on QTLs for *U. pisi* resistance in pea. Vijayalakshmi et al. (2005) and Rai et al. (2011) studied in a BC_1_F_2_ population the resistance to *U. fabae* Pers. de-Bary, the pathogen responsible for rust disease in pea in sub-tropical regions, reporting that it was governed by a major and a minor QTL identified in the LG 7 as *Qruf* (22.4–58.8% of phenotypic variation; flanked by SSR markers *AA446* and *AA505*) and *Qruf1* (11.2–12.4% of phenotypic variation; flanked by SSR markers *AD146* and *AA416*).

As mentioned above, several pea linkage maps based on different type of markers have been constructed. Most of these are gel-based markers and have limited ability to rapidly analyse a large number of marker loci. Although some of these limitations can be overcome by using specialized hardware such as high-throughput capillary electrophoresis equipment that can improve the ability of allelic discrimination, reproducibility, and speed, there are still limitations related to the sequential nature, the requirement to have DNA sequence information to expand the currently available marker toolkits and the high costs of use these marker technologies. Mainly this last factor has made the use of molecular markers directly in wild species impractical. In this scenario, the Diversity Arrays Technology (DArT) in combination with next-generation sequencing platforms (Kilian et al., 2012; Raman et al., 2014) known as DArTseq™, provides a good choice as a high throughput marker genotyping platform that can develop a relatively large number of polymorphic markers to build dense genetic maps with low-cost investments. The thorough coverage of the genome and the high-density genetic maps based on DArTseq™ technology increase the power of QTL detection (Thudi et al., 2011). Additional advantages of DArTseq™ technology are its suitability for polyploid species as well as the possibility of developing rapidly for virtually any genome. As a result, DArTseq-derived markers are currently used in more than 400 species (http://www.diversityarrays.com/), being very popular among crops with the non-sequenced genomes. The DArTseq markers include genome-wide profiling of a large number of SNPs and detection of insertion/deletion polymorphisms which is easily expandable for genetic scope (Kilian et al., 2012). So far, the DArT markers have been used in legumes for the mapping of pigeon peas (*Cajanus cajan* L.) (Yang et al., 2011), chickpeas (*Cicer arietinum* L.) (Thudi et al., 2011), lupins (*Lupinus albus* L.) (Vipin et al., 2013), common beans (*Phaseolus vulgaris* L.) (Valdisser et al., 2017), and soybeans (*Glycine max* L.) (Vu et al., 2015).

Therefore, the goals of this work have been the development of the first integrated high-density genetic linkage map of *P. fulvum* and the identification of QTLs of resistance to pea rust.

## Materials and methods

### Plant material

A mapping population of 84 F_7_ RILs derived from the cross IFPI3260 (resistant) × IFPI3251 (susceptible) was used to construct an integrated SilicoDArT + SNP + SSR + STS based linkage map and was screened for rust (*U. pisi*) resistance.

### Phenotyping analysis for rust resistance under field and controlled conditions

Seeds of the pea susceptible check cv. Messire and 84 F_7_ RILs from the cross *P. fulvum* IFPI3260 (resistant) × IFPI3251 (susceptible) segregating for rust resistance and their parental lines were sown in fields located at Córdoba (Spain) during the 2013/2014, 2014/2015 and 2015/2016 growing seasons. Each accession was represented by a single row of 1 m in which 15 plants were sown and which was separated from the adjacent row by 0.7 m. The assays for each season included three replications of each accession arranged in a completely randomized design. Plots were artificially inoculated at the end of March to ensure high and uniform levels of rust infection. Prior to field inoculation, stock spores of the local *U. pisi* isolate Up-Co01 conserved at −80°C were heat shocked at 40°C for 5 min and multiplied on susceptible pea cv. Messire. These seedlings were spray-inoculated with an aqueous urediniospores suspension (±1.0 × 10^5^ urediniospores ml^−1^) to which Tween-20 (0.03%, v: v) was added as a wetting agent and incubated for 24 h at 20°C in complete darkness and 100% relative humidity. Later, they were transferred to a growth chamber at 20°C with a photoperiod of 14 h of light and 10 h of darkness and a light intensity of 148 μmol m^−2^ s^−1^. After 2 weeks, the fresh urediospores were collected using a vacuum spore collection device and used for field inoculation. The plants were inoculated after sunset to benefit from the darkness and high relative humidity of the night. At maturity, disease severity (DS) and infection type (IT) were assessed. DS was visually estimated as the percentage of canopy covered by rust pustules. IT was assessed using the 0–4 scale of Stackman et al. (1962), where IT 0 = no symptoms, IT; = necrotic flecks, IT 1 = minute pustules barely sporulating; IT 2 = necrotic halo surrounding small pustules, IT 3 = chlorotic halo and IT 4 = well-formed pustules with no associated chlorosis or necrosis.

In addition, the response of the plant material was studied in seedlings under controlled conditions. Two seeds were sown in each pot (35 × 35 cm) with a 1:1 mixture of sand and peat. Three replicates were performed with 8 plants (4 pots) per accession and replicate in a completely randomized design. Seedlings were inoculated when the third leaf was completely expanded (± 12 days after germination). The inoculation was carried out by dusting the plants with the urediospores of *U. pisi* isolate Up-Co01 (2 mg spores pot^−1^) diluted in pure talc (1:10, v:v) using a spore settling tower. The plants were incubated for 24 h at 20°C in complete darkness and at 100% relative humidity. Then they were transferred to a growth chamber at 20°C with a photoperiod of 14 h of light and 10 h of darkness and 148 μmol m^−2^ s^−1^ of irradiance at plant canopy. DS and IT values were recorded 10 days after the inoculation as above mentioned. DS values measured in both field and controlled conditions were normalized by the arcsine transformation [y = arcsine (√DS)]. Pearson's linear correlations between rust resistance parameters were performed using Statistix (version 8.0; Analytical Software, Tallahassee, USA).

### DNA extraction and quantification

The eighty-four *P. fulvum* F_7_ RILs of the mapping population and the parents of the cross were grown under controlled conditions at IAS-CSIC. Around 1 g of the young leaf tissue from the 3rd to 4th node of each seedling was excised, immediately frozen in liquid nitrogen and stored at −80°C. Genomic DNA was isolated from the frozen leaves using a modified cetyltrimethylammonium bromide (CTAB)/chloroform/isoamylalcohol method (Doyle and Doyle, 1987). DNA quantification was performed by agarose gel electrophoresis (0.8 %), and it was adjusted to 50 ng/μl for DArT and SNP genotyping and to 5 ng/μl for SSR genotyping.

### Genotyping of individual DNA samples using DArTseq technology

A high-throughput genotyping method using the DArT-Seq™ technology at Diversity Arrays Technology Pty Ltd (Canberra, Australia) was implemented to genotype the F_7_ RIL population of *P. fulvum*. Essentially, DArT-Seq™ technology relies on a complexity reduction method to enrich genomic representations with single copy sequences and subsequently perform next-generation sequencing using HiSeq2000 (Illumina, USA). DArT-Seq detects both SNPs and presence–absence sequence variants, collectively referred to as DArT-Seq markers (Raman et al., 2014). DArT-Seq was optimized for *P. fulvum* by selecting the most appropriate complexity reduction method (*PstI-MseI* restriction enzymes). DNA samples were processed in digestion/ligation reactions as described by Kilian et al. (2012), but replacing a single *PstI*-compatible adapter with two different adapters corresponding to two different restriction enzymes (RE) overhangs. The *PstI*-compatible adapter was designed to include Illumina flowcell attachment sequence, sequencing primer sequence, and staggered, varying length barcode region. The reverse adapter contained the flowcell attachment region and *MseI*-compatible overhang sequence. Only “mixed fragments” (*PstI*–*MseI*) were effectively amplified in 30 rounds of PCR using the following reaction conditions: 1 min at 94°C for initial denaturation; 30 cycles each consisting of 20 s at 94°C for denaturation, 30 s at 58°C for annealing and 45 s at 72°C for extension; and finally a 7 min extension step at 72°C. After PCR, equimolar amounts of amplification products from each sample of the 96-well microtiter plate were bulked and applied to c-Bot (Illumina) bridge PCR followed by sequencing on Illumina Hiseq2000. The sequencing (single read) was run for 77 cycles. Sequences generated from each lane were processed using proprietary DArT analytical pipelines. In the primary pipeline, the FASTQ files were first processed to filter poor-quality sequences, applying more stringent selection criteria to the barcode region compared to the rest of the sequence. Thus, the assignments of the sequences to specific samples carried in the “barcode split” step were more consistent. Approximately 2,500,000 (±7%) sequences per barcode/sample were used in marker calling. Finally, identical sequences were collapsed into “fastqcall files.” These files were used in the secondary pipeline for DArT P/L's proprietary SNP and SilicoDArT (Presence/Absence Markers in genomic representations) (present = 1 vs. absent = 0) calling algorithms (DArTsoft14). The analytical pipeline processed the sequence data.

Parameters for the DArT marker assaying pipeline for quality control (Kilian et al., 2012) such as: (i) the reproducibility of 100%, (ii) the overall call rate (percentage of valid scores in all possible scores for a marker) over 95%, (iii) the polymorphic information content (PIC) between 0.3 and 0.5 and (iv) the *Q*-value (the logarithm of the minimum false discovery rate at which the test may be called significant) above 2.5 were used for selecting high-quality SilicoDArT and derived SNPs markers for genetic mapping.

### Genotyping with simple sequence repeats (SSRs), sequence tagged site (STS) and single-nucleotide polymorphism (SNP) markers

A set of 46 genic and genomic SSRs (15 fluorescently labeled SSRs and 31 M13-labeled SSRs) previously described by Loridon et al. (2005) was screened using the two parental lines and the 84 F_7_ RILs. A multiplex PCR (markers labeled with different dyes) was carried out to amplify SSRs following optimal PCR conditions previously published by Loridon et al. (2005). Then 2 μl of the PCR product were taken from each marker of the multiplex set and pooled together for simultaneous detection of the amplified alleles. Seven μl of formamide and 0.2 μl of fragment-size standard GeneScanTM 500 LIZ were added to the pooled PCR product and run on an ABI 3730 DNA genetic analyzer (Applied Biosystems). The data were collected automatically by the detection of the different fluorescences and analyzed using GeneMapper v4.0 software (Applied Biosystems).

In addition 10 STS markers reported by Gilpin et al. (1997) were surveyed for polymorphism using the protocol described by Barilli et al. (2010). When no polymorphism was detected, PCR products from both parents were digested with a range of restriction endonucleases which recognized 4- and 5- base sequences (BioLabs_inc_, Ipswich, MA, USA). A 0.2 μl aliquot of restriction enzyme, 12 μl of sterile water, and 2.5 μl of the buffer required for each enzyme were added to 10 μl of the PCR reaction, and the digestion was incubated overnight at 37°C. View of PCR products and gel images analyses were performed as described by Barilli et al. (2010).

Finally, 26 SNP markers developed using the BeadXpress Primer Design (Illumina, San Diego, CA, USA) (Deulvot et al., 2010) and previously described by Carrillo et al. (2014), were analyzed using the high-throughput genotyping method Illumina GoldenGate assay as described by Bordat et al. (2011).

### Linkage mapping and QTL mapping

The scores of all polymorphic DArTseq markers, SSR, STS, and SNP markers were converted into genotype codes (“A,” “B”) according to the scores of the parents. Linkage groups were obtained using the software JoinMap version 4.1 (Van Ooijen, 2006). The maximum likelihood mapping algorithm, which was optimized for constructing dense genetic maps using this software (Jansen et al., 2001), was first used for grouping all of the polymorphic markers. Then, the method of regression mapping (Haley and Knott, 1992) was used for map construction with approximately 1,000 markers with appropriate genetic distance and the marker position and the order of markers for three rounds to merge the tightly adjacent markers into bins. The markers in adjacent loci with genetic distance below 0.2 cM were classified into a bin during the first two rounds of mapping. Moreover, one marker with sequence information and with the least missing genotype from each bin was chosen as a “bin representative” for the next round of genetic mapping. For the last (the third) round of mapping, the makers in adjacent loci pairs with genetic distances below 0.1 cM were classified into a bin to avoid incorrect classification when the markers were decreased in the map. The Kosambi mapping function (Kosambi, 1943) was used to convert recombination frequencies into map distances, and only “Map 1” was used for further analysis. The LG maps of each chromosome were drawn and aligned using MapChart v2.3 (Voorrips, 2002). The threshold for the goodness of fit was set to ≤ 5.0, with a recombination frequency of < 0.4. Linkage groups were separated using a LOD score ≥ 3.0. Markers with a mean Chi-Squared value of recombination frequency > 4.0 were discarded. The DArT markers were named with the numbers corresponding to unique clone ID following Kilian et al. (2012).

Several previously described anchor markers were used to find the correspondence between *P. fulvum* and *P. sativum* linkage groups and assign them to pea chromosomes. For the same purpose, the sequences from DArT-seq-derived markers were compared with *Medicago truncatula* genomic backbone by using Phytozome v.12 (https://phytozome.jgi.doe.gov/pz/portal.html) to perform a synteny analysis using three parameters recently defined by Salse et al. (2009). These parameters increase the stringency and significance of BLAST sequence alignment by parsing BLASTX results and rebuilding HSPs (High Scoring Pairs) or pairwise sequence alignments to identify accurate paralogous and orthologous relationships. This analysis allowed searching for sequence similarity-based homology between legume species providing an alternative approach to finding correspondence between linkage groups.

QTL analysis for rust resistance was conducted using composite interval mapping (CIM) and multiple interval mapping (MIM) in MapQTL 6.0 package (Van Ooijen, 2011). Markers to be used as cofactors for CIM were selected by forward–backward stepwise regression. Significance thresholds of log of odds (LOD) corresponding to a genome-wide confidence level of *P* < 0.05 were determined for each trait using the permutation test of MapQTL 6.0 with 1000 iterations, according to Barilli et al. (2010). Skewness and Kurtosis coefficients were calculated following Lynch and Walsh (1997). The coefficient of determination (R^2^) for the marker most tightly linked to a QTL was used to estimate the proportion of the total phenotypic variation explained by the QTL.

## Results

### Rust resistance

**Figure 1:**
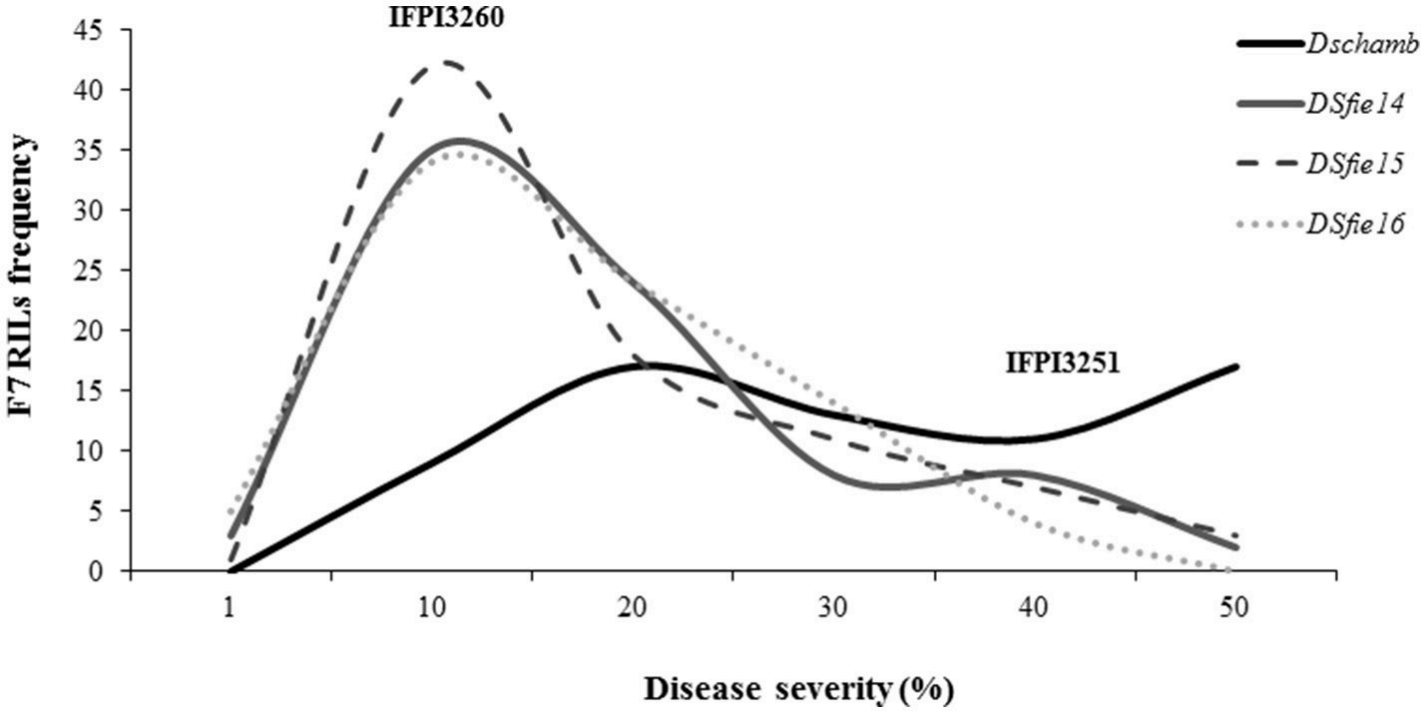
Frequency distribution of rust severity (%) among the F7 RIL progenies derived from the *P. fulvum* cross (IFPI3260 × IFPI3251) under controlled conditions (*DSchamb*) and under field conditions at Córdoba (Spain) in the growing seasons 2013/14 (*DSfie14*), 2014/15 (*DSfie15*), and 2015/16 (*DSfie16*).

*Pisum fulvum* accessions showed compatible interaction (high IT) against *U. pisi* although they differed in DS, confirming previous findings (Barilli et al., 2009a,b). Analysis of variance of each trial, either under field or controlled conditions revealed highly significant genotypic effects for DS resistance criteria among the RIL families (*P* < 0.05). The parental accessions showed contrasting DS responses, with IFPI3260 being highly resistant (*DS* < 5%) and IFPI3251 highly susceptible (*DS* > 30% in field and *DS* = 63% in controlled conditions) (Figure 1). Distribution of residuals after analysis of variance was normal in each year and tested conditions according to Shapiro-Wilk (*P* > 0.01). Variances of genotypes and replicates were homogeneous according to Bartlett's test (*P* > 0.05). The coefficient of Skewness in field conditions was of 0.86, 0.87, and 0.83 for seasons 2013/14, 2014/15, and 2015/16, respectively, which indicated that the population distribution tended to the resistance as the parent IFPI3260. Distributions of the estimated adjusted means are represented in Figure 1 for DS for each year and condition evaluated. DS values ranked between 0.75 and 45% under field conditions and 5–63.3% under controlled conditions. They did not differ from the normal distribution, confirming the quantitative inheritance of the partial resistance. In addition, transgressive segregants with increased resistance and susceptibility compared with the parentals were observed for DS resistance criteria over years and conditions. Pearson's linear correlation coefficients (Table 1) were highly significant between DS values evaluated within years in the field, as well as under controlled conditions.

**Table 1:** Pearson's linear correlation coefficient between the response to *U. pisi* (disease severity = DS) assessed in seedlings under controlled conditions and in adult plants under field conditions at Córdoba (Spain) during 2013/14, 2014/15, and 2015/16 growing seasons.

**Field conditions**	**Growth chamber**	**Field conditions (2015/16)**	**Field conditions (2014/15)**
2015/16	0.6572^***^		
2014/15	0.6741^***^	0.9561^***^	
2013/14	0.6841^***^	0.9374^***^	0.9188^***^

*** *Significant at P = 0.001*.

### Genotyping and linkage mapping

A total of 9,569 high-quality SilicoDArT and 8,514 SNPs markers were identified. Of these, a set of 8,538 SilicoDArT markers (89.2%) and 3,483 SNPs markers (40.9%) were selected for mapping after quality filtering. Thirty-five of the set of 46 SSRs markers previously described for *P. sativum* by Loridon et al. (2005) amplified in *P. fulvum*, which corresponded to a potential transferability rate of 76% for this marker type. However, only 12 markers were polymorphic. This meant that only 26.1% of the SSR markers tested showed utility for mapping in our population. The remaining 21 yielded non-specific amplification and were excluded from further analysis. The mapping dataset was complemented with 20 of the 26 SNP markers (76.9%) developed using the BeadXpress Primer Design (Illumina, San Diego, CA, USA) (Deulvot et al., 2010), as well as with 5 of the 10 STS markers (50%) reported by Gilpin et al. (1997), which provided reliably scorable polymorphism. As a result, 37 non-DArT-based markers were added to a set of 12,021 DArTseq derived markers that scored co-dominantely (Tables 2, 3). All the non-DArT-based markers mapped in the expected LGs according to previous publications (Loridon et al., 2005; Deulvot et al., 2010; Bordat et al., 2011; Carrillo et al., 2014; Tayeh et al., 2015). Markers were distributed across 7 LGs using LOD thresholds ranging from 3 to 10 and a recombination frequency (r) threshold <0.4 (JoinMap vs. 4) (Supplementary File 1). Each assigned group included at least two markers common to other published *P. sativum* genetic maps.

**Table 2:** Map features of IFPI3260 × IFPI3251 linkage map.

**Linkage group**	**Markers**				**Unique position**	**Distance (cM)**	**Average density**	**Larger gap**
	**DArT**	**SNP**	**Others**	**Total**				
LGI	1,238	552	2	1,792	163	307.408	1.886	7.143
LGII	1,488	570	5	2,063	181	349.478	1.931	8.451
LGIII	1,301	489	16	1,806	152	281.021	1.849	6.849
LGIV	1,218	542	4	1,764	121	219.543	1.814	6.757
LGV	1,159	453	4	1,616	139	265.575	1.911	13.043
LGVI	946	398	3	1,347	107	223.273	2.087	8.824
LGVII	1,188	479	3	1,670	131	231.154	1.764	7.246
Total	8,538	3,483	37	12,058	994	1,877.452	1.89	8.330

**Table 3:** Summary and description of the reference markers used to generate de *P. fulvum* composite map, including their linkage group assignment and position in the *P. fulvum* map and their correspondence to the *P. sativum* linkage groups and chromosomes.

**Marker ID**	**Marker type**	**Linkage Group (*P. fulvum*)**	**Position (cM)**	**Linkage Group (*P. sativum*)**	**References**
AA317	SSR	I	107.962	VII	Loridon et al., 2005
AA160	SSR	I	148.560	VII	Loridon et al., 2005
GAL_SNP1	SNP	II	39.164	V	Bordat et al., 2011
AB23	SSR	II	41.904	V	Loridon et al., 2005
P108-*Hyp188*	STS	II	85.639	V	Weeden, 2005
Kdsa_SNP3	SNP	II	144.864	V	Bordat et al., 2011
AD280	SSR	II	165.438	V	Loridon et al., 2005
sbe2_SNP3	SNP	III	7.058	III	Bordat et al., 2011
HO1_SNP1	SNP	III	8.573	III	Bordat et al., 2011
P202-*RSAI*	STS	III	12.986	III	Carrillo et al., 2014
fruct1A6Abipho_SNP1	SNP	III	15.843	III	Carrillo et al., 2014
OPT_SNP1	SNP	III	15.843	III	Carrillo et al., 2014
AA175	SSR	III	76.878	III	Loridon et al., 2005
AD57	SSR	III	112.152	III	Loridon et al., 2005
PsAP1_SNP3	SSR	III	118.974	III	Bordat et al., 2011
T-complex_SNP1	SNP	III	122.995	III	Carrillo et al., 2014
AA491	SSR	III	152.925	III	Loridon et al., 2005
rfs_SNP3	SNP	III	152.925	III	Bordat et al., 2011
M27-*TaqI*	STS	III	222.61	III	Bordat et al., 2011
Enod12B_SNP1	SNP	III	264.072	III	Bordat et al., 2011
AD174	SSR	III	279.632	III	Loridon et al., 2005
Bfruct_SNP4	SNP	III	281.021	III	Weeden, 2005
AD270	SSR	III	281.021	III	Loridon et al., 2005
P482-*HhaI*	STS	IV	132.165	II	Weeden, 2005
AA102	SSR	IV	150.136	II	Loridon et al., 2005
peptrans_SNP1	SNP	IV	193.57	II	Bordat et al., 2011
peptrans_SNP2	SNP	IV	193.57	II	Bordat et al., 2011
AA335	SSR	V	46.346	VI	Weeden, 2005
ARBA3199_567	SNP	V	165.754	VI	Carrillo et al., 2014
PsDHN1_124	SNP	V	194.37	VI	Bordat et al., 2011
PsDHN1_320	SNP	V	194.37	VI	Bordat et al., 2011
agps2_SNP3	SNP	VI	0.000	I	Bordat et al., 2011
ARBA8726_644	SNP	VI	7.247	I	Tayeh et al., 2015
subt_SNP2	SNP	VI	177.687	I	Bordat et al., 2011
P393-*HhaI*	STS	VII	146.03	IV	Weeden, 2005
GTPaseact_SNP1	SNP	VII	165.224	-	Unpublished results
gpt2_SNP1	SNP	VII	215.554	IV	Bordat et al., 2011

**Figure 2:**
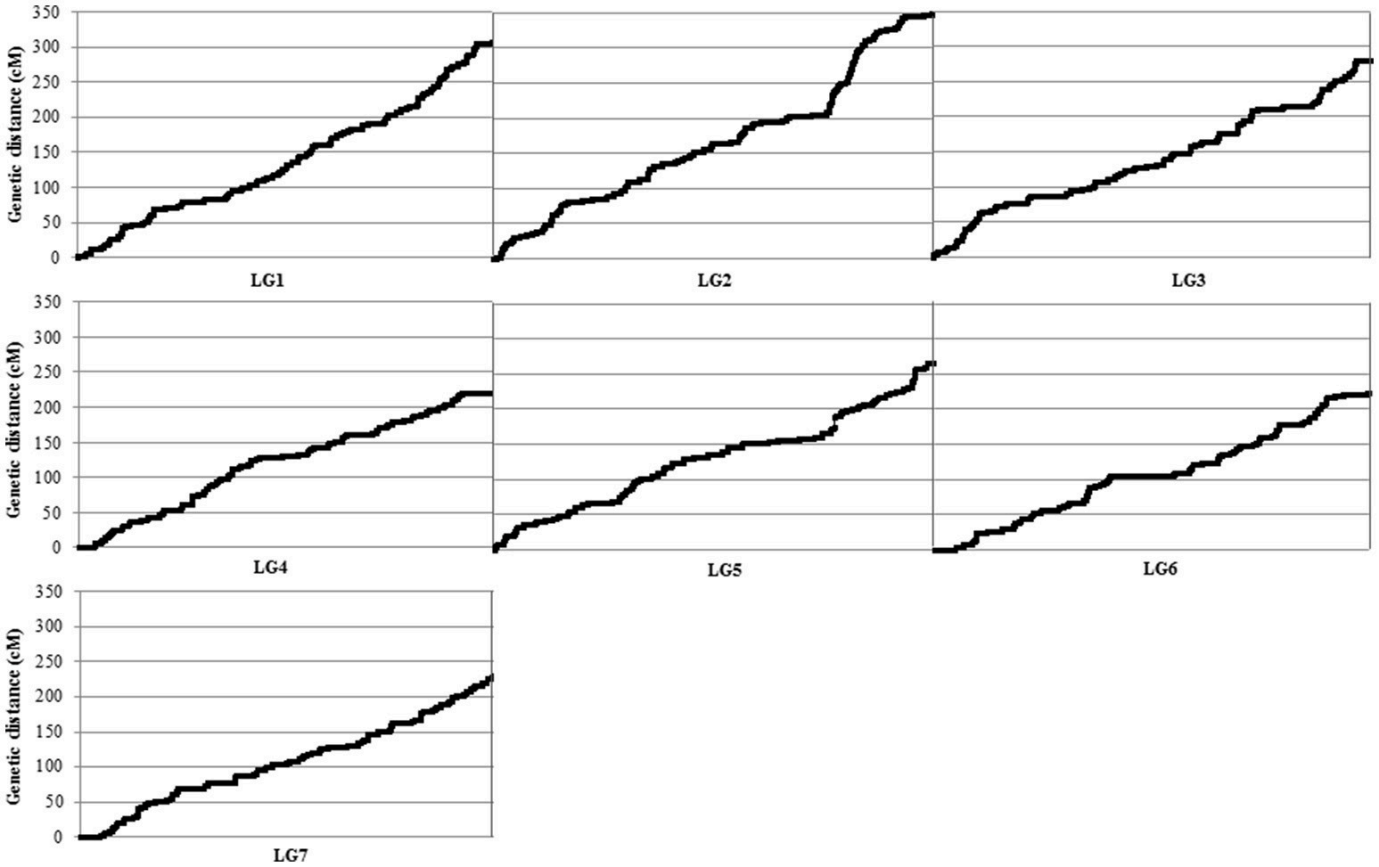
Distribution of the DArT-seq-based and no-DArT-seq markers within each linkage group (LG) forming the map derived from the cross between *P. fulvum* IFPI3260 × IFPI3251. The *x* axis shows the LG and the *y* axis shows the genetic distance (cM).

The newly constructed integrated genetic linkage map of *P. fulvum* covered a total length of 1877.45 cM, with an average density of 1.19 markers cM^−1^ and an average adjacent-marker gap distance of 1.85 cM (Table 2). The total number of mapped loci per linkage group ranged from 1347 on LGVI to 2068 on the LGII, and the average was 1,723 loci LG^−1^. The longest individual linkage group map was for the LGII (349.48 cM), the shortest was for the LGIV (219.54 cM) (Figure 2), and the average LG length was 268.21 cM. The density of markers in the individual linkage groups ranged from 1.76 markers cM^−1^ in the LGVII to 2.09 markers cM^−1^ in the LGVI. Map distances between two consecutive markers varied from 0 to 13.04 cM, while the gap average between markers varied from 1.66 cM in the LGVII and 2.05 cM in the LGVI (Table 2).

Thirty-seven previously described markers (Table 3), as well as 98 sequences from *P. fulvum* DArTseq-derived markers that were BLASted with *M. truncatula* genome (Supplementary File 2), allowed to define the correspondence between *P. fulvum* and *P. sativum* linkage groups and the pea chromosome assignment, as follows: 2 SSR markers (AA317 and AA160) as well as 20 DArTseq-derived markers linked the *P. fulvum* LGI to the *P. sativum* LG7; 5 previously reported markers, as well as 14 DArTseq-derived markers linked LGII to LG5; 16 previously reported markers, as well as 12 DArTseq-derived markers linked LGIII to LG3; 4 markers (P482-*HhaI*, AA102, peptrans_SNP1 and peptrans_SNP2) and 5 DArTseq-derived markers related LGIV to LG2; 4 previously reported markers (AA335, ARBA3199_567, PsDHN1_124, and PsDHN1_320) and 10 DArTseq-derived markers related the LGV to the *P. sativum* LG6; 3 SNP markers (agps2_SNP3, ARBA8726_644, and subt_SNP2) and 18 DArTseq-derived markers linked the LGVI to the *P. sativum* LG1; finally, 3 previously mapped markers (P393-*HhaI*, GTPaseact_SNP1 and gpt2_SNP1) and 10 DArTseq-derived markers showed that the LGVII corresponds with the LG4. The LGVI displayed an inverted markers order compared to Bordat et al. (2011) and Carrillo et al. (2014). Pea chromosome assignment following Ellis and Poyser (2002) was reported in Table 3.

### QTL mapping

**Figure 3:**
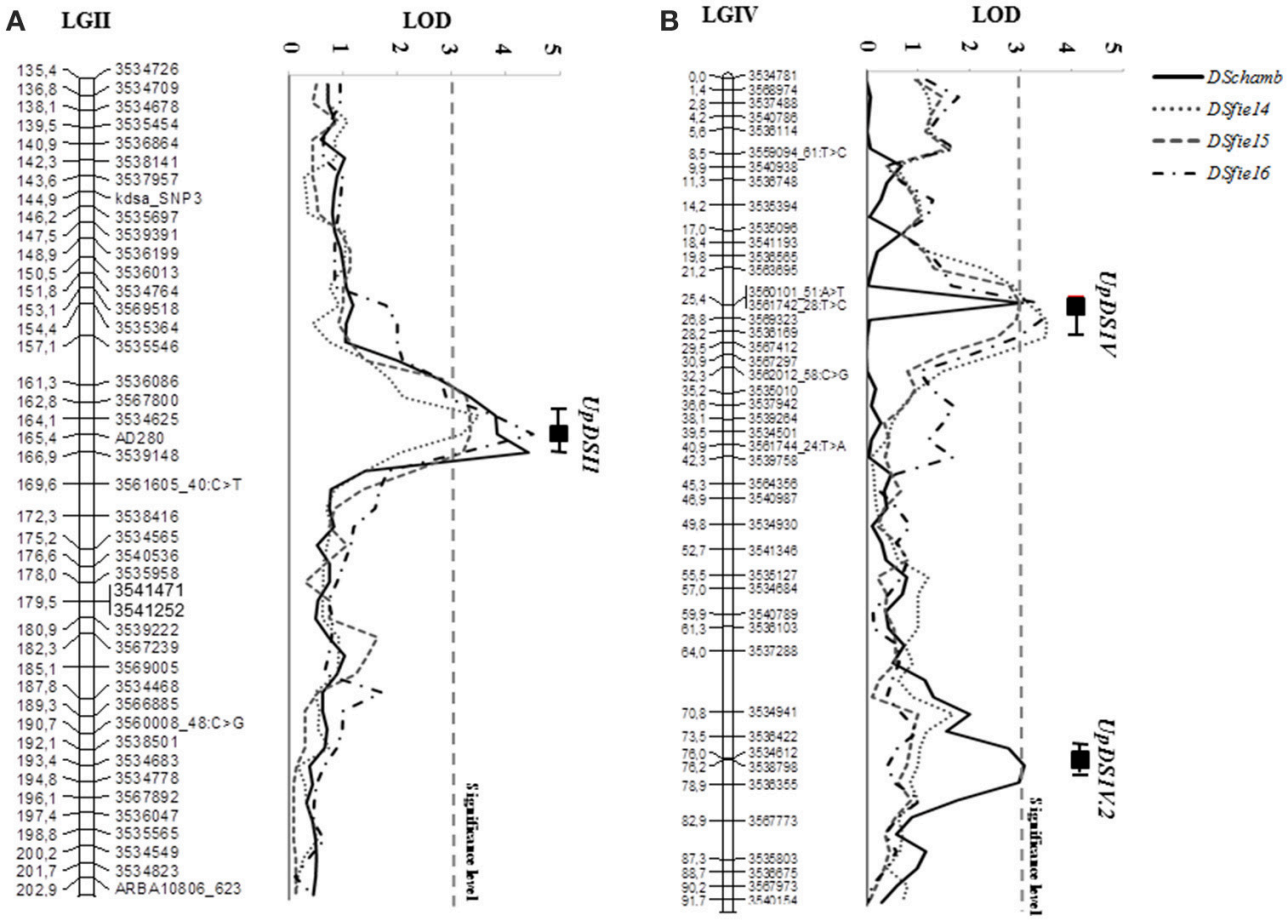
Likelihood plots of the consistent quantitative trait loci (QTLs) for seedling and adult plant leaf rust resistance assessed under controlled conditions and field conditions for the linkage groups (LGs) II **(A)** and IV **(B)** of the genetic map of the IFPI3260 × IFPI3251 RIL population using MapQTL. Significant LOD thresholds were detected based on 1,000 permutations. Absolute positions (in cM) of the molecular markers along LGs are shown on the vertical axes. DSfie14, DSfie2015 and DSfie2016: % of disease severity at Córdoba (Spain) during seasons 2013–2014, 2014–2015, and 2015–2016, respectively; DSchamb: is the % of disease severity under controlled conditions.

The scoring of disease severity under controlled and field conditions allowed the identification of genomic regions involved in pea rust resistance. Quantitative trait loci analysis using CIM and MIM methods revealed 2 genomic regions associated with the resistance of adult plants to *U. pisi* under field conditions located in the LGs II and IV. Results were consistent along all the growing seasons considered. The QTL *UpDSII* showed a LOD score range of 3.28–4.51 and explained 20.7–29.2% of rust severity variation at the adult plant stage (Table 4, Figure 3A). *UpDSII* was localized between 163.7 and 164.2 cM from the beginning of the LGII, between the derived DArT marker 3567800 and the previously known SSR marker AD280. The distance to the left and to the right flanking markers ranged between 0.9–1.4 and 1.2–1.7 cM, respectively. The second consistent QTL, *UpDSIV*, showed a LOD score ranging from 3.00 to 3.47 and explained 19.8–22.1% of the variation of the rust severity at the adult plant stage (Table 4, Figure 3B). *UpDSIV* was localized between 25.4 and 26.7 cM from the beginning of the LGIV, between the derived DArT markers 3563695 and 3569323 (for DSfie14 and DSfie15), and 3560101_51:A>T and 3536169 (for DSfie16) (Table 4). The distance to the left and to the right flanking markers ranged between 1.5–4.7 and 0.9–1.5 cM, respectively. Both QTLs explained together 43.8, 40.5, and 51.3% of pea rust severity variation in *DSfie14, DSfie15*, and *DSfie16*, respectively.

**Table 4:** Position and effects of the quantitative trait loci (QTLs) for resistance to *U. pisi* in *P. fulvum* IFPI3260 × IFPI3251 RIL population based on the percentage of disease severity (DS%) scored in the field during three growing seasons (2013/14, 2014/15, 2015/16) at Córdoba (Spain) as well as under controlled conditions, applying composite interval mapping (CIM) and multiple interval mapping (MIM) in MapQTL 6.0.

**QTL^a^**	**LG^b^**	**Trait name^c^**	**Position^d^**	**Left marker**	**Right marker**	**LOD^e^**	**Add^f^**	**R^2^^g^**
*UpDSII*	II	DSfie14	164.2	3567800	AD280	3.63	−4.12	22.8
		DSfie15	163.7	3567800	AD280	3.28	−4.39	20.7
		DSfie16	164.1	3567800	AD280	4.51	−3.04	29.2
		DSchamb	165.4	3534625	3539148	4.42	−13.11	28.2
*UpDSIV*	IV	DSfie14	25.4	3563695	3569323	3.42	−4.41	21.0
		DSfie15	25.9	3563695	3569323	3.00	−3.22	19.8
		DSfie16	26.7	3560101_51:A>T	3536169	3.47	−4.12	22.1
		DSchamb	25.4	3563695	3569323	3.05	−3.25	19.9
*UpDSIV.2*	IV	DSchamb	76.0	3536422	3538798	3.00	−2.93	14.1

a *QTL that extend across single one-log support confidence intervals were assigned the same symbol*.

b *LG linkage group*.

c *DSfie14, DSfie15 and DSfie16: disease severity (%) under field conditions (Córdoba, Spain) during growing season 2013/14, 214/15 and 2015/16, respectively; DSchamb: disease severity (%) under controlled conditions*.

d *Peak QTL position (cM)*.

e *LOD the peak LOD score*.

f *Add the additive effect*.

g *R^2^ proportion of phenotypic variance explained by the respective QTL (%)*.

These results were also confirmed under controlled conditions after performing the QTL analysis for resistance to *U. pisi* in wild pea seedlings. In fact, the consistent QTL *UpDSII* (LOD = 4.42) located in the LGII was localized at 165.4 cM from the beginning of the LGII (peak QTL position coinciding with the SSR marker AD280), between the derived DArT markers 3534625 and 3539148. The distance to the left and the right flanking markers was of 1.3 and 1.5 cM, respectively (Figure 3A). The second consistent QTL, *UpDSIV*, was also located at 25.4 cM from the beginning of the LGIV (LOD = 3.05) between the derived DArT markers 3563695 and 3569323, as previously found (Figure 3B). The distance to the left and to the right flanking markers was of 4.2 and 1.4 cM, respectively and explained 19.9% of the phenotypic variation (Table 4). In addition, a third QTL associated with rust resistance in controlled conditions was found in the LGIV (named *UpDSIV.2*) (Table 4). *UpDSIV.2* showed a LOD score of 3.0 and explained 14.1% of the phenotypic variation. The distance to the left and to the right flanking markers was of 2.8–0.2 cM, respectively. All the three QTLs explained together 62.2% of pea rust severity variation in *DSchamb* (Table 4). Sequence information of all flanking markers is listed in Supplementary File 1.

The resistance-enhancing allele originates from the resistant parent IFPI3260 as shown by the negative value of the additive genetic effect (ranging from −13.11 to −2.93) (Table 4).

## Discussion

Genetic linkage maps are highly valuable tools for the identification of candidate genes/QTLs that can be used in map-based cloning and marker-assisted breeding programs. Dense genetic maps based on sequence-derived markers, such as diversity arrays technology (DArTseq™) markers accelerate the process of fine mapping and map-based cloning of genes/QTLs. DArT-derived markers have been extensively used in recent years to build comprehensive genetic maps in legumes such as chickpea, pigeonpea, peanut and soybean (Vu et al., 2015; Ahmad et al., 2016). To the best of our knowledge, the present study is the first extensive published linkage map reported in wild pea (*P. fulvum*). The RIL mapping population was generated from a cross between two parental lines belonging to the *P. fulvum* specie showing excellent genetic diversity and providing a high frequency of polymorphisms of great utility for the construction of the linkage map. In fact, a total of 12,021 (66.5%) markers were found polymorphic in the panel of 84 RILs. In addition, the selected wild pea DArTseq markers showed a good average polymorphic information content (PIC) value of 0.42 (values ranging from 0.30 to 0.50), which indicated that those markers should be considered useful or informative (Kilian et al., 2012). Therefore it is very evident that DArT markers can be developed and typed quickly and economically compared to other marker technologies (Ahmad et al., 2016).

The map of *P. fulvum* constructed in this work is composed by 7 LGs that cover a total length of 1,877.4 cM and an average adjacent-marker distance of 1.8 cM. It includes 12,021 polymorphic DArT markers that together with 37 previously published markers of diverse nature (SNP, SSR and STS working as anchor markers) has been used as a bridge allowing us to combine our linkage map with recently published *P. sativum* consensus maps (Loridon et al., 2005; Bordat et al., 2011; Carrillo et al., 2014; Tayeh et al., 2015) and determine the orientation of the 7 LGs found. The comparison of these bridge markers of our IFPI3260 × IFPI3251 *P. fulvum* map with the previous *P. sativum* maps has revealed a general high consistency of marker order with only minor exceptions, as it was the change of the order of some marker within the same LG, or the case of the LGVI that showed an inverted order of anchoring markers with respect to those previously reported in *P. sativum* (Loridon et al., 2005; Carrillo et al., 2014). Such differences in the order of the markers among *P. fulvum* and *P. sativum* linkage maps were not unexpected, since genetic mapping only provides an indication of the relative positions and genetic distances of the markers among themselves (Loridon et al., 2005) and structural rearrangements of the chromosomes could be relatively common between *P. fulvum* and *P. sativum* (De Martino et al., 2000). Nonetheless, the inconsistency of the position on the map of these few SSRs could be explained by the presence of closely linked DArT loci (Kilian et al., 2012; Lan et al., 2017).

Over 85% of the 12,061 mapped markers showed marker–marker linkage tendency (in groups of at least two markers), which collapsed into 994 bins. These markers were likely to represent mostly gene-rich regions, as DArT method of complexity reduction targets the hypomethylated regions of the genome. This is consistent with the observations found in the genetic maps of many other species such as chickpea and rapeseed (Thudi et al., 2011; Raman et al., 2014) as an example. Mapping of DArT array markers to the Eucalyptus reference genome using the unique sequence tag of each marker has suggested that *PstI*-based DArT markers are predominantly at the low-copy, gene-rich regions (Petroli et al., 2012). However, most of the observed clustering of marker could be explained by the limited resolution of our mapping population because its size was small (below 100 individuals).

The total genetic length of the presented map is 46.3% larger than the map (1,283.3 cM) previously constructed using the F_2_ IFPI3260 × IFPI3251 RIL population in which 146 markers including 144 RAPD and 2 STS (Barilli et al., 2010) were mapped, but smaller than the maps of *P. sativum* published so far. In fact, the total lengths of some of them has even reached 2,416 (Timmerman-Vaughan et al., 2004) or 2,555 cM (Sudheesh et al., 2015). The map length obtained in the present study suggests that high-density and high-quality DArT genotyping data obtained after minimizing genotyping errors and missing data have contributed greatly to a better estimation of the distance among the markers.

The distribution of the markers was reasonably uniform along the map. In fact, the marker density of each individual linkage group ranged from 1 marker/1.764 cM (LGVII) to 1 marker/2.087cM (LGII), with a total average of 1 marker/1.89 cM with is a 9-fold increase compared to the previous published *P. fulvum* map (Barilli et al., 2010). It has been suggested that uniformly distributed loci every 10 cM over the entire genome is effective for MAS and QTL identification (Stuber et al., 1999). In this contest, the high level of genome coverage achieved in this map will be particularly useful for selecting markers for use in whole-genome breeding strategies and for saturating genomic regions of interest in other mapping populations. In addition, although around 12,000 markers were identified in this study, more markers are available for further work since more than 18,000 clones from *P. fulvum* were obtained. Thus, the *P. fulvum* DArT platform has been shown to be useful for the application in genome-wide screening for QTL discovery. It could also be expected to demonstrate its usefulness for recurrent parent background recovery in marker assisted backcrossing, for isolation of genes via map based cloning, comparative mapping and genome organization studies. The availability of better saturated molecular maps that can be achievabled using DArTSeq technology, as an example, will certainly provide breeders and geneticists with a much-desired tool for identifying genomic regions of interest, which in turn will increase the efficiency of marker-assisted breeding according to Ren et al. (2015).

The present work is also the first report of a QTL analysis for phenotypic traits on a high-density linkage map in a F_7_ derived RIL population of *P. fulvum* using the DArTseq^TM^ technology. Pea rust caused by *U. pisi* is a major challenge to pea growers in temperate regions but complete resistance to this disease has not been identified so far (Barilli et al., 2009b,c). However, high incomplete resistance has been reported in wild *Pisum* accessions, especially in *P. fulvum* accession IFPI3260 that shows good levels of resistance at both seedling and adult plant stages and to different isolates of *U. pisi* (Barilli et al., 2012), being a suitable source of resistance to this disease. In the present study, no hypersensitive resistance reaction to *U. pisi* was observed. Neither the resistant parent nor the RILs were free of rust infection in the 3-year field experiments at Córdoba (Spain) and neither under controlled conditions. The resistance traits scored showed a continuous distribution in the RIL population, which indicated the quantitative nature of their inheritance. This is in agreement with the previous findings in which partial resistance was not associated with hypersensitive response against *U. pisi*. (Barilli et al., 2009a,b,c) or *U. fabae* (Singh and Sokhi, 1980; Vijayalakshmi et al., 2005; Chand et al., 2006; Ren et al., 2015) in *Pisum* spp. Most of the RIL families revealing a level of resistance skewed toward low disease severity rates which suggested that the combination of both parental lines could enhance the level of resistance provided by the resistant accession IFPI3260.

Two major and a minor QTLs distributed over two linkage groups were found to be associated with the DS%. Two consistent QTLs, *UpDSII* and *UpDSIV*, were located in the LGs II and IV, respectively. A minor QTL named *UpDSIV.2* was also located in the LGIV. This was not in agreement with the previous QTL analysis performed on the F2 RIL population derived from the same cross, where a single genomic region associated with the resistance to *U. pisi* was identified and a QTL (*Up1*) explaining more than 60% of phenotypic variation under controlled conditions was described on LG 3 (Barilli et al., 2010). This discrepancy between both analyses could presumably be due to the advancement in the generation of the segregating populations, the number and nature of markers involved and/or software processing. The previous QTL *Up1* was located in a genomic region poorly saturated in markers and the RAPD markers associated were not robust and transferable (Barilli et al., 2010).

The present study has demonstrated that the genetics of pea rust resistance is somewhat complex and controlled by several QTLs. The QTLs *UpDSII* and *UpDSIV* were consistently identified by DS% in both adult plants over 3 years at the field and in seedling plants under controlled conditions. Whenever they were detected, their contribution to the total phenotypic variance was substantially high (19.8–29.2). Previous studies in legumes have shown good correlations between rust disease assessments in seedlings under controlled conditions and adult plants in the field, suggesting the existence of genetic factors that control the effectiveness of the resistance at different developmental stages and environments (Singh et al., 2015; Rai et al., 2016). For *U. fabae* resistance in *P. sativum*, Rai et al. (2016) reported that major QTLs *Qruf* and *Qruf2* were common to all the resistance traits evaluated including the disease severity in the seedling and in the adult plants in different environments.

The minor QTL *UpDSIV.2* was environmentally specific and was only detected for DS% in seedlings under controlled conditions. It accounted more than 14% of the phenotypic variation studied. Minor QTLs are observed quite frequently in disease resistance studies, but they are prone to inconsistent expression (Pilet-Nayel et al., 2002; Cobos et al., 2005). The three QTLs (*UpDSII, UpDSIV*, and *UpDSIV.2*) showed all negative additive effect. This indicated that the resistance alleles came from the resistant parent, which is supported by the presence of transgressive segregants with a lower disease severity.

Our results have indicated that, taken together, the identified QTLs have explained a very high percentage of the phenotypic variance throughout the population (40.5–51.3% of pea rust severity variation in the field and 62% in controlled conditions), suggesting that an efficient selection could be possible with a few markers tightly linked to the resistant QTLs. The SSR marker AD280 and the DArT-derived marker 3567800 were located at less than 1.5 cM, delimiting a region fairly close to *UpDSII*. Similarly, the DArT-derived markers 3563695 and 3569323 were located at less than 5 cM of the QTL *UpDSIV*, as well as the markers 3536422 and 3538798 to the QTL *UpDSIV.2*. For the QTLs *UpDSII* and *UpDSIV* the peak LOD could move 1 cM depending on the year or the conditions of rust evaluation, with some minor changes in the linked markers. This is not unusual, since molecular mapping of disease resistance QTLs in legumes has often revealed the same QTL located in comparable genomic regions but having different markers closely linked depending on how the disease intensity was scored or if the evaluation were carried out in different years (Hamon et al., 2011; Carrillo et al., 2014; Rai et al., 2016). Although the mapping population of our study was evaluated in three different years and different conditions, the genomic positions of the QTLs were comparable in all the cases not without observing that the markers closely linked to the QTLs varied in some of the cases.

Disease resistance genes are commonly organized in complex clusters or loci (Loridon et al., 2005). These regions of the linkage maps are often rich in genes conferring resistance to different pathogens and/or to different races of the same pathogen. After connecting our *P. fulvum* map with the previously published *P. sativum* linkage maps (Loridon et al., 2005; Bordat et al., 2011; Carrillo et al., 2014), we have found that the QTLs *MpV.2* and *MpII.1* conferring resistance to *Didymella pinodes* (Carrillo et al., 2014) as well as the QTLs *Ae-Ps5.2* and *Ae-Ps2.2* conferring resistance to *Aphanomyces euteiches* (Hamon et al., 2011) were co-localized with *UpDSII* and *UpDSIV*, respectively. This suggested that the genomic regions of the LGs II and IV of *P. fulvum* where *UpDSII* and *UpDSIV* are likely to control disease resistance and to harbor clusters of disease resistance genes in pea. The identification of such genomic regions involved could be useful in resistance breeding programs through marker assisted selection (MAS).

In this sense, the additional advantage offered by DArTseq™ markers is that they can be readily converted into PCR-based markers (Fiust et al., 2015) in cases where there are still no low-cost markers that closely flank a potential QTL. Also, there is a great possibility of identifying candidate genes that could be used in the foreground selection of favorable alleles since DArTseq produces a large number of markers within gene regions. The identification of potential candidate genes is the first step to identify the genes that control *U. pisi* resistance in pea. However, additional studies such as functional analysis are needed to finally validate the role of the gene in resistance. The suitability of these genes as candidates for resistance to *U. pisi* would facilitate an efficient MAS.

## Conclusions

The results obtained from the present study indicated that DArTseq™ provides high-quality markers that can be used to construct dense genetic linkage maps for plants even when no sequence information is available. A total of 37 polymorphic species-specific SSR, STS and SNP markers and 12,021 DArTseq™ based markers were used to develop a reasonably well saturated genetic linkage map of the interspecific *P. fulvum* RIL population derived from the cross between IFPI3260 × IFPI3251, allowing a precise and fine QTL mapping of important phenotypic traits related to *U. pisi* resistance in wild pea. The rust resistance loci identified in the LGs II and IV have been a novel report. The expression of the resistance to *U. pisi* in *P. fulvum* originated from the resistant parent IFPI3260. DArTseq™ will be very useful both in pea breeding programs and in parallel ongoing projects in legumes.

## Author contributions

EB and DR designed the experiments. EB performed the RIL population development and the rust evaluations. EB and MC carried out the *P. fulvum* DNA extractions as well as the SSRs and STSs evaluation in the RIL population. EC performed the SNPs analysis in the RIL population and critically reviewed the manuscript. EB and MC carried out the QTL analysis and wrote most of the manuscript. DR contributed to the interpretation of results and writing of the manuscript. AK and JC performed DArT analysis, marker selection as well as established the synteny between *P. fulvum* and *M. truncatula*. AK and JC also contributed to critical reading.

### Conflict of interest statement

The authors declare that the research was conducted in the absence of any commercial or financial relationships that could be construed as a potential conflict of interest.

## Supplementary material

Supplementary File 1 Summary and description of the DArT-derived markers used to generate the *P. fulvum* map including marker identity, sequence description, linkage group assignment in the map and the position in the linkage group.

Supplementary File 2 Summary and description of the trimmed sequences of the *P. fulvum* DArT markers with correspondence to *Medicago truncatula* genome and their correspondence to *P. sativum* linkage groups.
